# Understanding the lived experience of lung cancer: a European social media listening study

**DOI:** 10.1186/s12885-022-09505-4

**Published:** 2022-04-30

**Authors:** Ana Rodrigues, Jyoti Chauhan, Alexandros Sagkriotis, Sathyaraj Aasaithambi, Michele Montrone

**Affiliations:** 1 Medical Oncology, Instituto Português de Oncologia do Porto Francisco Gentil, EPE, Porto, Portugal; 2 Novartis Healthcare Pvt Ltd (H.A.), Hyderabad, India; 3 Novo Nordisk Health Care AG, Zürich, Switzerland; 4 Medical Thoracic Oncology Unit, IRCCS Istituto Tumori “Giovanni Paolo II”, Bari, Italy

**Keywords:** Lung cancer, Non-small cell lung cancer, Social media, Social media listening, Real-world evidence, Patient journey, Quality of life

## Abstract

**Background:**

Social media platforms are increasingly being used by stakeholders to generate, access, and share health-related information and experiences. Lung cancer is the most common cancer, impacting > 2 million patients globally. This observational study utilized a social listening approach to analyze social media trends and gain insights into stakeholder perceptions of lung cancer.

**Methods:**

This social media study retrospectively collated data from open access blogs, forums, and social networking sites. Social media posts were collected between June 2019–May 2020 from 14 European countries. Using social media aggregator tools, posts comprising lung cancer and non-small cell lung cancer-specific terms were extracted. Manual and automated relevancy algorithms filtered the extracted information to provide the relevant dataset. This contextualized dataset was further mined to generate the final data for analysis.

**Results:**

Of 1360 conversations analyzed, 42% were generated by patients/caregivers and 14% by healthcare professionals (HCPs). A majority of patients were 51–70 years old (approximately 50%) and 91% (*n* = 500/550) had late-stage cancer. Treatment (35%) and disease awareness (30%) were among the most discussed topic of the patient journey. Although the overall treatment sentiment was neutral, chemotherapy was the treatment type with the highest associated negative sentiment (28%); fewer negative sentiments were associated with immunotherapy (9%) and targeted therapy (2%), due to perceptions of longer survival outcomes and fewer side effects. In conversations that discussed clinical endpoints, “survivability” and “overall survival” (47 and 30%, respectively; *n* = 539) were most frequently mentioned by stakeholders. HCPs mostly used technical terms, whereas patients and caregivers used colloquial terms such as “getting rid of cancer”. Emotional wellness was identified to have a huge impact on quality of life in lung cancer. Delay or treatment cancellations due to COVID-19, lack of effective treatments and funding, and lack of empathy by physicians emerged as the key unmet needs among patients/caregivers.

**Conclusions:**

Social listening proved to be an effective tool to explore stakeholders’ perceptions and their key unmet needs, typically not available in published literature or databases, and provides HCPs with valuable insights into the distress, doubts, and needs of lung cancer patients and caregivers.

**Supplementary Information:**

The online version contains supplementary material available at 10.1186/s12885-022-09505-4.

## Introduction

Lung cancer is one of the most common malignancies worldwide and is the leading cause of cancer mortalities, with an estimated incidence of > 2 million cases and approximately 1.8 million deaths in 2020 [1]. Lung cancer is also a leading cause of cancer-related deaths in Europe, accounting for approximately 384,000 deaths in 2020 [2].

The majority of lung cancers (~ 85%) are classified as non-small cell lung cancer (NSCLC) [3]. Small cell lung cancer (SCLC) represents 13% of all newly diagnosed cases of lung cancer worldwide, or > 180,000 cases/year [4]. Despite advances in early detection and standard treatment, approximately 70% of NSCLC patients present with locally advanced or metastatic disease [5], with a low 5-year overall survival rate ranging from 4 to 17% [6].

Although smoking is a well-known risk factor associated with lung cancer, 10–25% of people who develop lung cancer are never-smokers and its cause cannot be definitively associated with environmental risk factors such as exposure to secondhand tobacco smoke, radon, air pollution, and asbestos, or with having had lung disease previously or an inherited genetic susceptibility [7, 8].

NSCLC is often associated with targetable genetic alterations or predictive biomarkers such as epidermal growth factor receptor (*EGFR*) mutations, anaplastic lymphoma kinase (*ALK*) rearrangements, c-ros oncogene 1 (*ROS1)* rearrangements, v-raf murine sarcoma viral oncogene homolog B1 (*BRAF)* V600E mutations, N-methyl-N′-nitroso-guanidine human osteosarcoma transforming gene (*MET)* exon 14 skipping mutations, rearranged during transfection (*RET)* rearrangement, Kirsten rat sarcoma viral oncogene homolog (*KRAS*), and neurotrophic tyrosine receptor kinase (*NTRK*) gene fusions [9, 10]. Another biomarker, programmed cell death-1 receptor (PD-1), which allows cancer cells to evade T cell inflammatory activity, has been established as a key immune biomarker [10]. Therapeutic strategies have been designed to inhibit these signaling pathways, among which are monoclonal antibodies and tyrosine-kinase inhibitors [10–18].

The treatment decisions for NSCLC are based mainly on the stage (extent) of the cancer, but other factors, such as a person’s overall health and lung function, as well as certain traits of the cancer itself, are also important. For instance, the 5-year relative survival rate for metastatic NSCLC is approximately 6% in patients receiving historic cytotoxic chemotherapy regimens compared to targeted therapies or immune checkpoint inhibitors (ICIs), with 5-year survival rates ranging from 15 to 50%, depending on the drug target [10]; however, not all treated patients benefit from these advanced therapies. Patients with advanced or metastatic NSCLC are being offered systemic therapy based on the presence of targetable genetic alterations or PD-L1 expression [10, 12].

Lung cancer is often associated with poor quality of life (QoL), due to the symptoms related to advanced disease, e.g., cough, dyspnea, pain, asthenia, anorexia, and hemoptysis. The adverse effects of treatments, particularly chemotherapy, also negatively impacts QoL [19]. Moreover, given the established relationship between smoking and lung cancer, patients who have smoked may feel stigmatized or guilty after diagnosis and more pessimistic about their illness and likely outcomes, all of which may have adverse implications for QoL [20].

Social media channels are online (often mobile) platforms that support the creation and exchange of user-generated content [21]. Social media platforms are projected to have 2.46 billion users worldwide [22]. A systematic review that analyzed clinical outcomes of social media use in chronic disease revealed that Facebook, blogs, and Twitter were the most popular social media platforms examined, and that cancer was the most frequent chronic condition investigated [23]; lung cancer was noted to be second most prevalent cancer discussed on Twitter after breast cancer [22]. Studies reporting social media use by patients and caregivers highlighted that online support groups, social networking sites, discussion forums, and micro-blogs (a type of blog which allows users to post small pieces of digital content like pictures, video, or audio on the internet) dominate the research literature, with cancer being the focus of 11.3% of the identified studies [24].

Our current understanding of how individuals affected by lung cancer use social media to describe their experiences is limited; however, existing studies do provide insights into how lung cancer stakeholders use social media, and have started to elucidate the importance of different platforms as channels for patient engagement and support, or as potential research data sources [21, 25, 26]. The insights from a social media listening (SML) approach in lung cancer are particularly important for treating physicians, as often patients feel stigmatized and avoid talking much about their experiences in person. Moreover, social media insights are not available in any other real-world databases (RWD) such as registries and electronic health records (EHRs).

Building on this, the present study set out to explore and understand what lung cancer stakeholders, including patients, caregivers, and HCPs, are discussing on social media platforms. This study used an SML approach to analyze social media trends and gain qualitative insights into stakeholders’ perceptions regarding burden of illness, epidemiology, patient characteristics, treatment patterns and compliance, QoL, predictors of outcomes, and effectiveness/safety.

## Methods

### Search strategy and data collection

Data regarding lung cancer and NSCLC-specific terms were collected retrospectively for 12 months from June 2019 to May 2020 across 14 European countries (the United Kingdom [UK], Spain, France, Switzerland, Belgium, Germany, Austria, the Netherlands, Italy, the Nordic countries, and Portugal) in the following languages: English, Spanish, French, German, Dutch, Italian, Portuguese, and Nordic languages (Swedish, Norwegian, Danish, and Finnish). Data were collected from open access blogs, forums, and social networking sites (including Twitter, public Facebook, and YouTube); SocialStudio [27] and Talkwalker [28] were used for UK and non-UK data collection, respectively. Search strings were built in each language to identify lung cancer-related posts/conversations, and Boolean operators (AND, OR) were used to combine individual keywords within the search strings (Supplementary Table 1). Conversations are defined as any relevant social media posts relating to stakeholders (patients, caregivers, family and friends, HCPs) experiences on key themes of the NSCLC, as specified in this study.

### Data analysis

A 3-tier technique was used to identify relevant data, with random sampling procedures generating the final dataset for analysis (Supplementary Fig. 1). Conversations containing lung cancer and NSCLC-specific terms were extracted using search strings and social media aggregator tools. The information was filtered to a contextualized dataset by automated relevancy algorithms (containing keyword-based relevancy algorithms) and manual review against pre-defined criteria (Supplementary Table 2).

The initial raw dataset underwent a relevancy check through in-house automation (conducted with the help of thorough taxonomies), to exclude categories such as buy/sell content, animal content, job postings, market research reports, and link duplicates, and to ensure that conversations provided relevant insights into the patient journey stage and other patient-centric topics. The automated methodology allows large amounts of data to be analyzed quickly and efficiently to dismiss any irrelevant posts. However, this approach poses a potential risk of missing out on some relevant posts as the nuances of human expression may not have been captured in some/all cases.

The output from automated relevancy check is then analyzed manually to check if any other irrelevancy has crept in. The final cleaned dataset is then contextualized by assessing the content for the possibility of answering at least one research question in scope. Once the contextualized data sample is ready, relevant posts were categorized by channel type (open access blogs, forums, or social networking sites) and, where possible, categorized by stakeholder (patients, caregivers, family and friends, HCPs, and others, based on the language used in the post, e.g., “my wife just started a cure with Tagrisso for an adenocarcinoma at stage IV”. A deep-dive analysis was performed on the filtered data sets to further analyze insights and themes relating to stakeholder perceptions of multiple aspects of the lung cancer and NSCLC experience, including disease burden (including QoL), epidemiology and patient characteristics, real-world effectiveness (including treatment choice and patient-reported outcomes [PROs]), treatment patterns and compliance (including treatment sequence and discontinuation), real-world safety (including treatment sentiment and side effects), and predictors of favorable outcomes. The manual analysis, however, could create some biases depending on how an individual analyst perceives the content being analyzed; also, in regard to the judgement on sentiments, it brings in the possibility of an analyst perception of what is negative, positive, or neutral. However, to eliminate such risks, the analyzed data were validated through multiple quality checks to help remove any biases.

In this analysis, the majority of outcomes were reported with numbers/percentages, however, some results were incremental insights that were inferred from conversations by the analysts, hence, no percentages or numbers were associated with these outcome measures.

### Patient confidentiality

All data utilized and presented in the present SML study were obtained from publicly accessible sources without accessing password-protected information. The pharmacovigilance approval was secured for the conduct of this study. All personal identifiers were removed from the downloaded data to anonymize the information. The pharmacovigilance approval by Novartis’ adverse event and safety reporting team was secured for the conduct of this study. This program was registered on One Registry under the registry ID: GSMR 2631 (V1)/1P1R).

## Results

### Overview of analyzed social media posts

The data universe extracted from the initial search using predefined keywords consisted of 261,952 social media posts. Of these, 22,596 posts were identified as contextualized data relevant to the study objectives. Further data filtering by iterative random sampling yielded 1360 posts containing records from key stakeholders which were then used for further analysis (Supplementary Fig. 1).

**Fig. 1:**
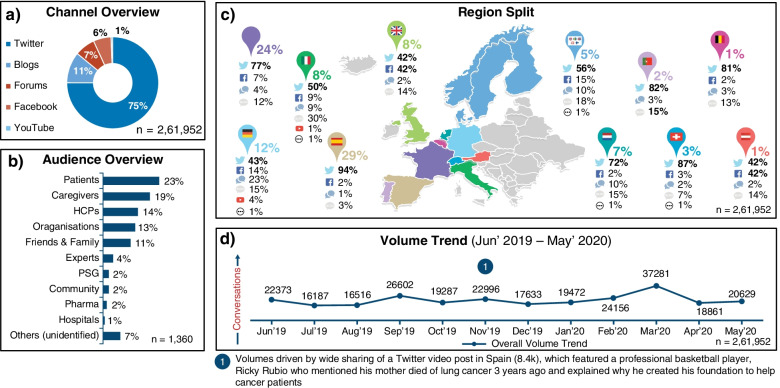
Data source and origin of relevant posts. **a** Data source of relevant posts; **b** Stakeholder overview based on analyzed data; **c** Country of origin of relevant posts; **d** Data volume trend. HCPs, healthcare professionals; PSG, patient support groups.  Twitter;  Facebook;  Blogs;  YouTube;  Forums

Overall, lung cancer had 242,000 mentions across the 14 European countries. From the total extracted data (*n* = 261,952), Twitter was the primary channel for discussion (75%), followed by blogs (11%) and forums (7%); (Fig. 1a). In the country-wide split of social media posts (Fig. 1c), compared to Twitter, Facebook has emerged as the predominant channel in Austria and the UK (42% each), while blogs were commonly used in Italy (30%). From the analyzed data (*n* = 1360 posts), patients (23%) and caregivers (19%) were the primary stakeholders, followed by HCPs (14%); (Fig. 1b). The volume trend from June 2019 to May 2020 as shown in Fig. 1d, peaked in March 2020 with 37,281 posts.

The country-wide key conversation themes are presented in Supplementary Fig. 2. Across countries, awareness generation around lung cancer, mortality, and risk-related factors such as smoking, as well as the importance of early detection, were more prominently discussed in social media conversations. Topics such as treatment (e.g., treatment experiences and side effects of the treatment) and research updates were some of the other common conversation drivers.

### Key demographic features of the social media population

**Fig. 2:**
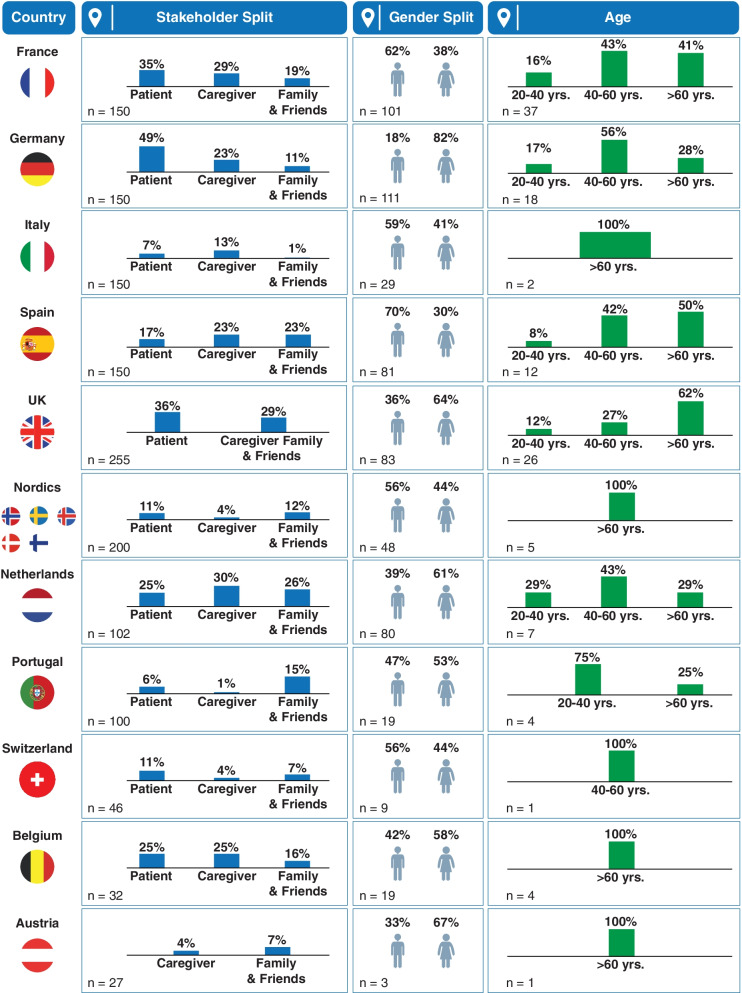
Key stakeholder demographics by country

The key stakeholder demographics across countries are presented in Fig. 2. Most patients were 51–70 years old (44.4%; *n* = 52/117), with the highest share of social media use in France (54%; *n* = 20/37) and Germany (56%; *n* = 10/18). Of all 583 gender-identified conversations, the overall visibility of female stakeholders (54%) was higher than males (46%). However, the prevalence of male patients was higher in Spain, France, Italy, and the Nordics. The number of conversations around smoking history was very low and smoking status was mostly unidentified. In Italy, smoking was the only cause of lung cancer discussed, with little awareness about other risk factors.

### Patient journey in lung cancer

**Fig. 3:**
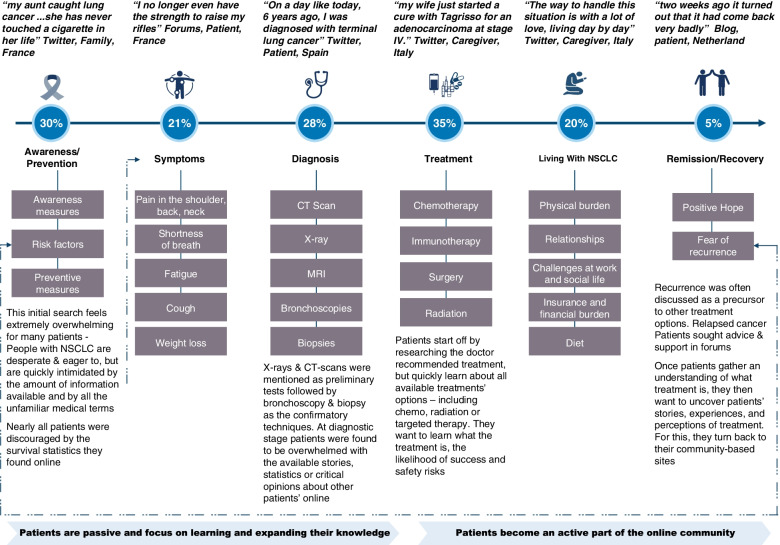
Patient journey analysis: Overall segments. NSCLC, non-small cell lung cancer

The study provided key insights into the patient journey of those living with lung cancer. While analyzing the social media conversations we observed that, during the early stages of the patient journey, patients/caregivers tended to passively observe online forums to increase their disease knowledge, only getting involved by asking questions and being thankful for any response. As their journey progressed, patients/caregivers were more actively involved by answering questions and sharing experiences online, thereby becoming advocates or active members of the community. Of 1360 analyzed posts, treatment (35%), awareness/prevention (30%), diagnosis (28%), and disease symptoms (21%) were the most frequently discussed themes (see Fig. 3). A number of posts also discussed topics such as cancer type and stage, metastatic sites, and genetic evaluation (Supplementary text and Supplementary Fig. 3).

### Experiences of lung cancer treatment

**Fig. 4:**
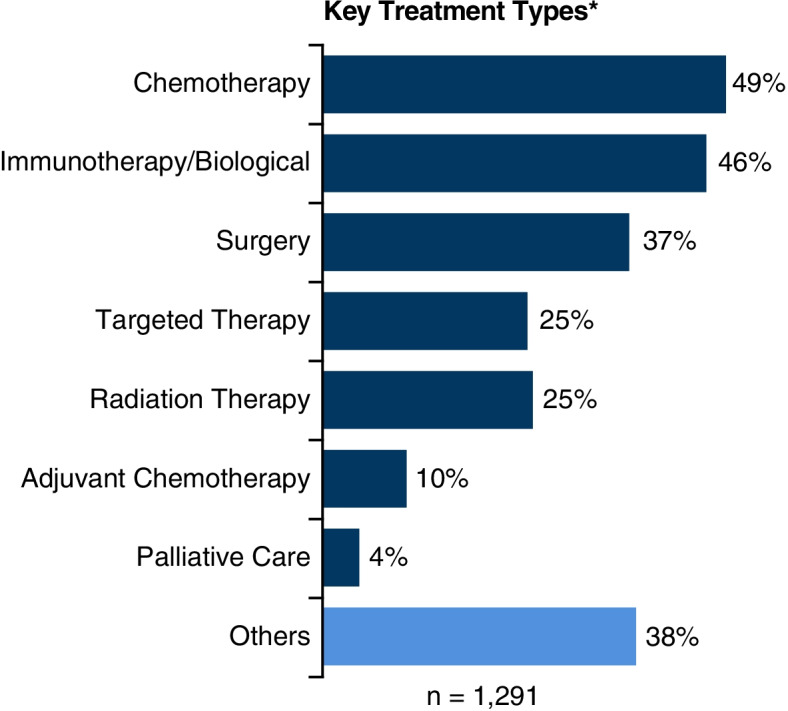
Treatment types – Overall. *Total % may be > 100% due to mention of multiple themes in posts

In terms of key treatment types (*n* = 1291), the most frequently discussed among stakeholders were chemotherapy (49%) and immunotherapy (46%), followed by surgery (37%),targeted therapy (25%), and radiation therapy (25%), as seen in Fig. 4; around 10% of patients also mentioned adjuvant chemotherapy. Palliative care (4%) was mentioned with regard to incurable cancer stages.

The overall sentiment toward treatments was neutral, as stakeholders described their journey and treatment options in a neutral manner (Supplementary Fig. 4). While chemotherapy had the highest share of negative perceptions (12% positive, 28% negative; *n* = 318); immunotherapy and targeted therapy were more positively perceived (47% positive, 9% negative; *n* = 276 and 47% positive, 2% negative; *n* = 139, respectively); sentiments towards surgery and radiation therapy were polarized (Supplementary Fig. 4).

**Fig. 5:**
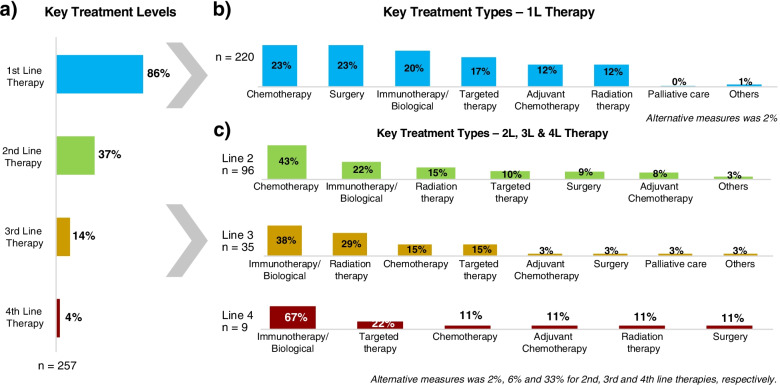
Treatment levels by treatment pattern. **a** Key treatment levels; **b** Key treatment types – 1 L therapy; **c** Key treatment types – 2 L, 3 L, and 4 L therapies. 1 L, first line; 2 L, second line; 3 L, third line; 4 L, fourth line

Around 19% (*n* = 257) of the treatment-related conversations mentioned the line of treatment or treatment sequence; in these conversations, patients (35%), caregivers (25%), and HCPs (12%) were the key contributors (Fig. 5a). While HCPs explicitly referred to lines of therapy, patients/caregivers often used terms relating to the treatment pattern, rather than referring to “lines of therapy”.

First-line therapy (1 L) was most prominently discussed in treatment-level conversations (86%; *n* = 220) (Fig. 5a). Chemotherapy (23%) was predominantly discussed as a 1 L therapy in most of the countries. Immunotherapy (20%) and targeted therapy (17%) were also mentioned as better alternative 1 L treatments in advanced lung cancer settings. Surgery (23%) was mentioned positively as the tumor was diagnosed in a curative setting, as shown in Fig. 5b.

Discussion on second line (2 L) therapy was comparatively low (37%; *n* = 96) and the options mentioned were chemotherapy (43%), immunotherapy (22%), radiation (15%), or targeted therapy (10%) (Fig. 5c).

**Fig. 6:**
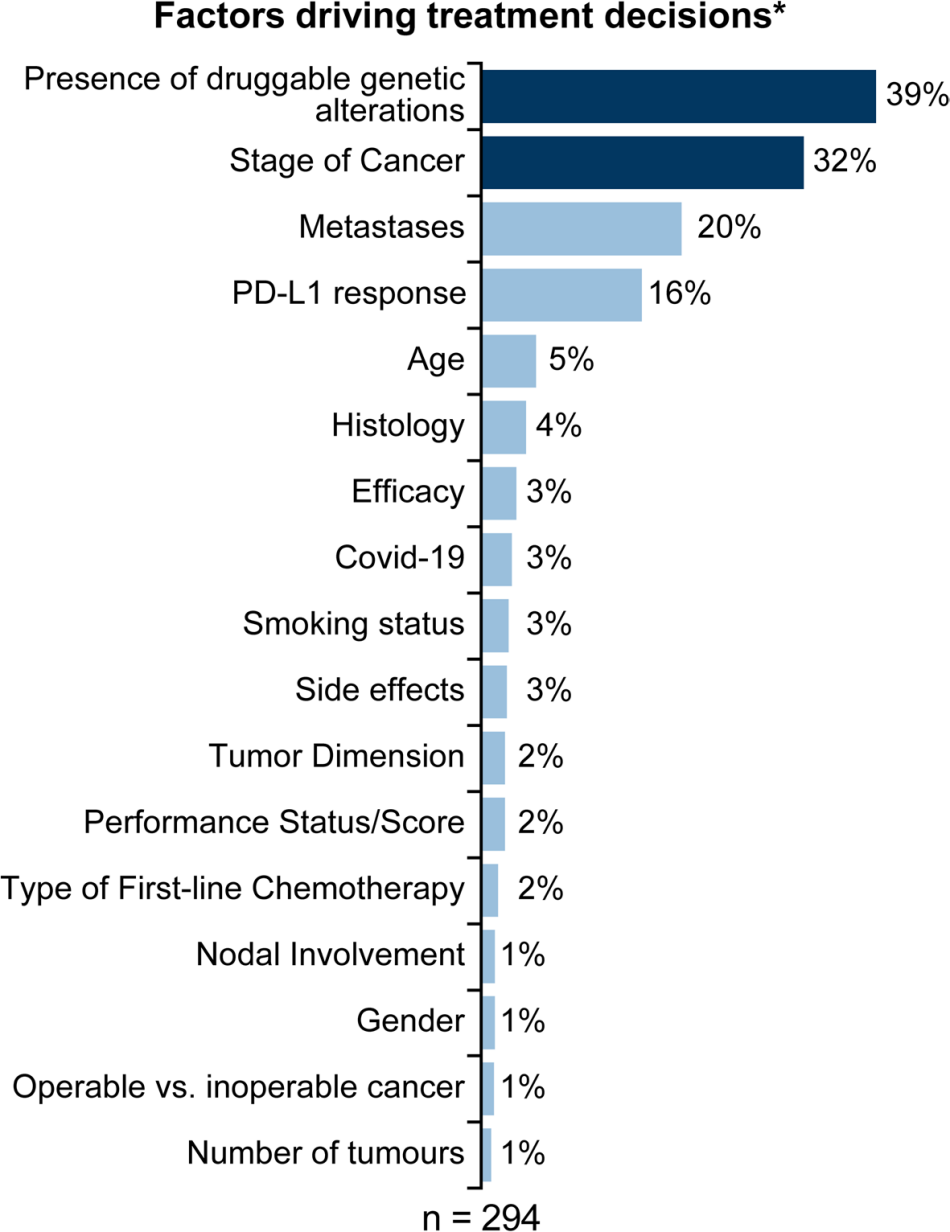
Key factors driving treatment decisions^*^. ^*^Total may be > 100% because multiple themes could be mentioned in posts. PD-L1, programmed death-ligand 1

The key factors driving treatment decisions among HCPs (*n* = 294) were presence of druggable genetic alterations (39%), stage at diagnosis (32%), metastases (20%), and PD-L1 expression (16%); (Fig. 6). The most frequently discussed druggable genetic alterations (≥5%; *n* = 219) were *EGFR* (54%), *ALK* (23%), *ROS1* (8%), *KRAS, MET*, and *RET* (5% each). While cancer stage was discussed as having an impact on the curability and survivability of patients, an advanced cancer stage limited treatment options and drove decisions toward palliative care.

Based on the site and spread of metastasis, either combination treatment approaches were applied, or certain options (e.g., surgery) were eliminated. The occurrence of metastases after a course of treatment led to treatment change to a more aggressive treatment strategy. In the Netherlands, PD-L1 was discussed as a prognostic factor for immunotherapy efficacy.

As expected, targeted therapy was mostly linked with mutation-related mentions (66%), with both HCPs/experts and patients being familiar with the terminology and potential benefits, followed by immunotherapy (19%), chemotherapy (16%), and adjuvant chemotherapy (11%); (Supplementary Fig. 5).

**Fig. 7:**
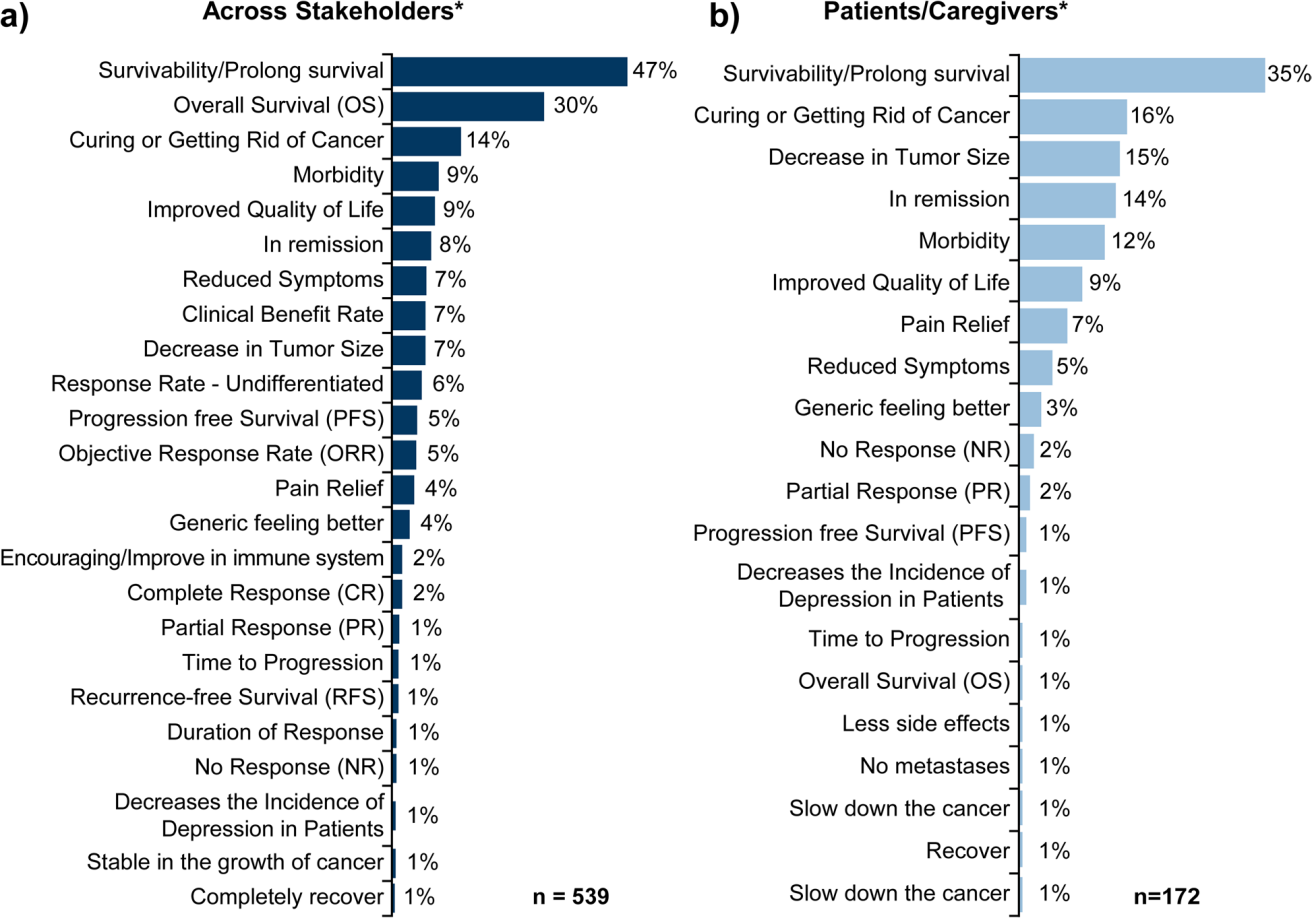
Clinical endpoints. **a** Across stakeholders; **b** Patient/Caregivers. *Total may be > 100% because multiple themes could be mentioned in posts

In posts that discussed disease endpoints (*n* = 539), survivability/prolonged survival (47%), and overall survival (30%) were the 2 most common clinical endpoints discussed by stakeholders. When analyzed by patients/caregivers (*n* = 172), the most frequently discussed endpoints were survivability/prolonged survival (35%) and curing/getting rid of cancer (16%); (Fig. 7).

For patients/caregivers, efficacy was of greatest importance and patients looked for a reduction in tumor size, symptom relief, improved QoL, and prolonged survival (Supplementary Fig. 6). Technical terms were mostly used by HCPs and organizations when referring to research studies, while patients/caregivers used terms related to efficacy (e.g., reduced symptoms, getting rid of cancer, improvement in QoL, and being in remission).

Discussions on treatment discontinuation (*n* = 45), highlighted the absence of treatment options, patients’ exhaustion, and intolerable side effects impacting QoL to be the main reasons for discontinuation and treatment change. While relapse, metastasis, and lack of desired effects/inefficacy were the key factors that led patients to switch to another treatment type, discovering metastasis drove a change to more intense treatment.

### Impact on QoL

**Fig. 8:**
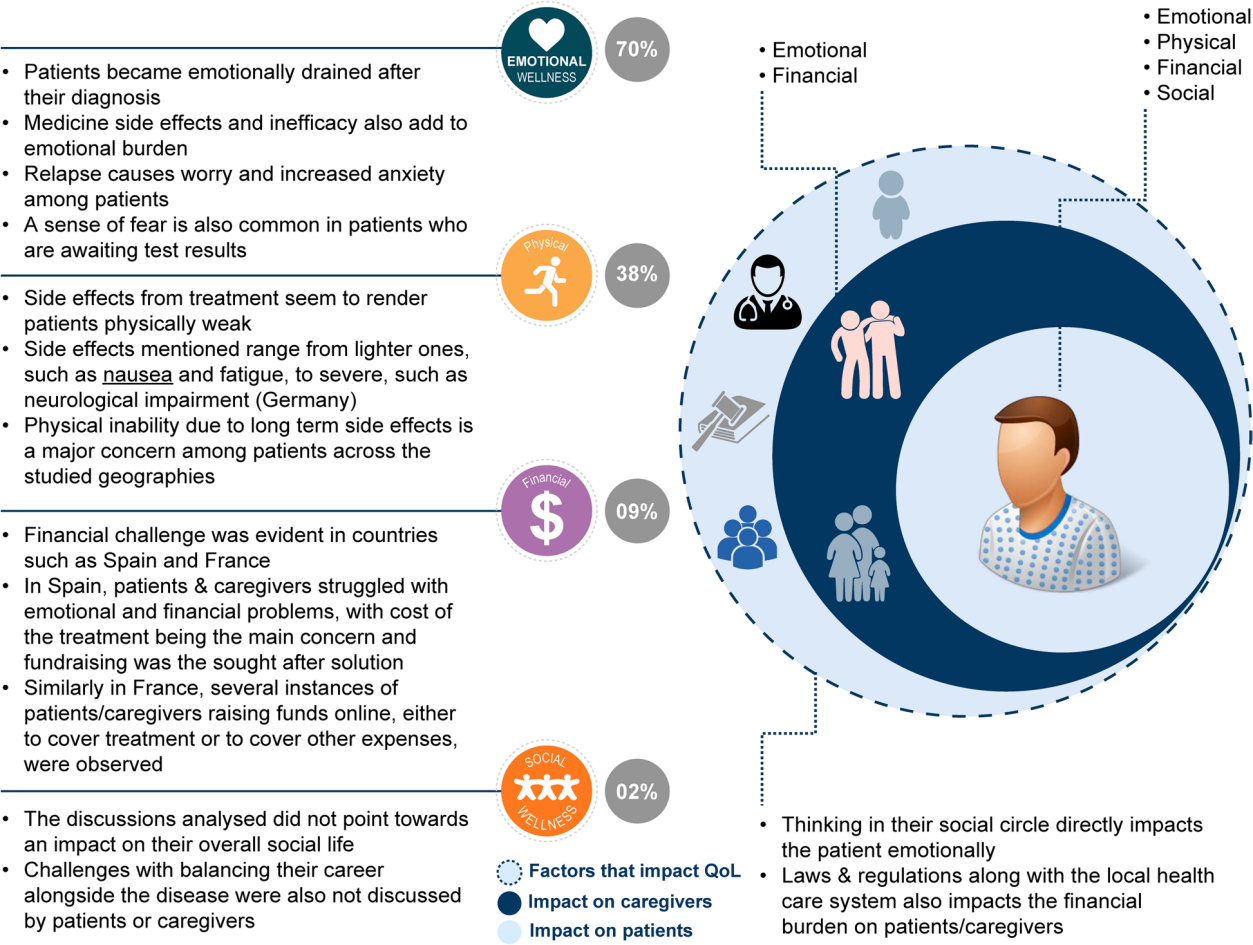
Impact on QoL. QoL, quality of life

Around 7% (100/1360) of the total conversations highlighted the impact on QoL, and 9% (48/539) of the conversations about clinical endpoints discussed QoL as a treatment goal. Emotional wellness was discussed in 70% of conversations across the patient journey, including diagnosis, treatment, and relapse (Fig. 8).

Physical challenges (38% of conversations) were mainly due to the adverse effects of treatment (primarily chemotherapy). Germany had the highest proportion of side effect-related conversations (40%, *n* = 38/94).

Financial challenges (9% of conversations) were evident in countries such as Spain and France, where some patients and caregivers raised funds to cover treatment costs and/or other expenses.

Social wellness was mentioned in 2% of conversations and analysis of these conversations did not point toward an impact on patients’ overall social life or to any challenges with balancing career alongside disease management (Fig. 8).

### Stigma associated with lung cancer

Feelings of stigma around smoking history were common, with patients feeling they were blamed for their condition. In most conversations, smoking status was not revealed, probably due to the stigma attached to it.

Several countries including France and Finland perceive lung cancer to be a self-inflicted condition and raise questions about providing healthcare support and treatment. In the Netherlands, a young adult patient wanted to raise awareness of genetic mutations and the fact that anyone could get lung cancer, not just smokers. In Finland, patient support groups, experts, and HCPs have worked to raise awareness and remove any associated stigma, while in other Nordics countries, never-smokers have tried to raise awareness and remove the stigma that might contribute to late detection and a lack of funding for treatment.

### Discussions around the impact of coronavirus disease 2019 (COVID-19)

Around 6% (85/1360) of the conversations analyzed mentioned COVID-19 with respect to its impact on NSCLC treatment (Supplementary Fig. 7a). Treatment delay and cancellation was the key effect from COVID-19, especially for patients receiving chemotherapy due to the lack of available ICU beds or resources and challenges in ensuring patient safety. A patient in Germany mentioned preferring to postpone her chemotherapy and only continue with immunotherapy, to avoid further compromising her immune system.

Infection concerns were caused by being vulnerable and the inability to find the requisite personal protective equipment (PPE) kits, failure to adhere to COVID-19 safety requirements during treatment administration, and lack of aftercare. In the Netherlands, patients reported premature release from hospital and raised concerns of missed aftercare and inadequate home support. SARS-CoV-2-positive patients with lung cancer were also reported in multiple countries, including Spain and Portugal.

### Other key unmet needs

As well as the COVID-19-related unmet needs, poor communication by HCPs and the lack of effective treatments caused frustration among patients and caregivers (Supplementary Fig. 7b). Patients and/or caregivers, particularly in France, Spain, the UK, and Germany, reported that HCPs lacked empathy or communicated insufficient information. Patients reported feeling abandoned when HCPs were “rushed” or “cold” during consultations, leading them to look for additional information online and, in the worst situations, resulting in them doubting HCPs’ competence and treatment decisions.

Running out of treatment options weighed heavily on patients.. In Italy and the Nordic countries, social disparity impacted treatment and caused late detection of lung cancer.

Insurance coverage and prejudice against lung cancer was also discussed in France, the Netherlands, and the Nordic countries. Targeted therapy was not covered by insurance in Norway, and patients/friends raised funds online to cover treatment or other expenses in France. In Finland, questions were raised as to whether patients should receive treatment coverage for what was perceived to be a self-inflicted disease.

## Discussion

This observational study demonstrated that social listening is a powerful tool that can be used to explore different elements of stakeholder perceptions, providing valuable insights that are not typically available in published literature or RWD. Using social media trends, we sought to explore stakeholders’ perceptions, preferences, and knowledge levels about various aspects of their lung cancer experience. This analysis identified differences in treatment goals and language used by patients/caregivers and HCPs; and highlighted patients’ desire to increase public awareness and access to information, and for greater physician empathy. The emotional burden of lung cancer has emerged as the leading cause impacting QoL in patients/caregivers, followed by the physical impacts. Patient interactions with social media evolve over time, from passive observer to active participant, possibly reflecting the emotional and educational journey. SML studies utilize publicly available information on digital platforms, however, they pose unique ethical challenges since individuals do not formally consent to the use of their posted data in the research. Although there is little current guidance on the lack of consent or anonymity of participants in social media research, a general recommendation is to ensure that data collected answers specific research questions and that data should be presented in a way that avoids identifying participants [29]. The data presented in our SML study were obtained from publicly accessible sources without accessing password-protected information or revealing personal identifiers.

The qualitative analysis of 1360 relevant posts showed that, in majority of posts, stakeholders’ discussions were related to treatment and diagnosis, causes and risk factors, QoL, and unmet needs. Patients/caregivers wanted to generate awareness around some of the less understood topics, such as the causes of lung cancer and advancements in NSCLC treatment and diagnostic methods. They talked about their treatment experiences, the side effects of treatment, and how it impacted their QoL. These findings are in line with previous studies suggesting that lung cancer patients are interested in discussing risk factors, prevention, and diagnosis of lung cancer [30, 31]. Another social media study also revealed that most lung cancer-relevant tweets focus on treatment and the use of pharmaceutical and research interventions, followed by awareness-raising, and prevention/risks [25].

Multiple insights were gathered from this SML study compared to PROs in clinical trials which were typically limited to treatment-related effects on QoL and survival [32, 33]. Within the context of the patient journey, treatment was the most discussed topic, followed by causes and diagnosis. Chemotherapy was the most frequently discussed treatment option, predominantly used as a 1 L therapy alone or combined with immunotherapy. However, it was also the most negatively perceived for causing more side effects (e.g., pain, exhaustion), which affected QoL, as shown in previous studies [19, 34].

Over the last decade, randomized controlled trials (RCTs) for targeted therapies or ICIs in the 1 L treatment of advanced NSCLC have demonstrated significant clinical efficacy [13–18], leading to accelerated, widespread adoption of their use as standard treatments in the advanced NSCLC setting [10, 12]. The present SML study also showed that information was shared about immunotherapy and targeted therapy being used as alternative 1 L treatment options in advanced stages of lung cancer. These therapies were mostly discussed as being effective and were perceived positively among patients/caregivers due to perceptions of longer survival and limited side effects. Our findings are consistent with previous studies reporting better PROs and QoL measures with immuno- and targeted therapies versus chemotherapy, due to less frequent adverse effects [32, 35]. Furthermore, surgery was seen as effective in the early stage but the cost and physical suffering from a complicated surgery drove negativity. France had high negative mentions of radiation therapy due to the side effects such as loss of taste, radionecrosis, and weakness.

Treatment discontinuation occurs due to disease progression, lack of efficacy, unacceptable side effects, or patient exhaustion. Treatment discontinuation was discussed more frequently in the Netherlands and the Nordic countries as an inevitable choice when patients had exhausted all the treatment options or experienced significant side effects. In Germany, a patient discontinued chemotherapy due to significant side effects and was shifted to immunotherapy, which proved to be effective. In the UK, side effects and the spread of cancer to other organs were the main reasons for switch and discontinuation of the treatment. There were also a few posts regarding the terminal stage when the treatment no longer worked and palliative care or a hospice was the last option.

A prospective cohort study highlighted that patients with lung cancer defined treatment success as increasing their survival along with maintaining QoL and functionality [36]. This is consistent with the findings from this SML study, where survivability and improved QoL parameters were considered the most commonly discussed clinical endpoints by lung cancer patients/caregivers and HCPs. In contrast, a systematic literature review on patient preferences found that patients often consider life extension to be more important than QoL or undesirable side effects [37]. Additionally, patients and caregivers generally used generic terms related to efficacy, i.e., reduction in symptoms, getting rid of cancer, improvement in QoL, and being in remission, to describe clinical endpoints, while HCPs/experts used technical terms with regard to research and studies, which revealed the differences in the language used by patients/caregivers and HCPs.

A high level of emotional impact from lung cancer was identified as the most frequently discussed topic among stakeholders. A study evaluating patients’ self-reported outcomes also found a strong negative relationship between QoL and anxiety, depression, and lung cancer stigma [38, 39]. Our findings accord with those of previous studies [38–40] demonstrating the strong stigma associated with a smoking history. These studies have also shown that this stigma can negatively impact QoL and even interfere with patients’ willingness to seek healthcare [39], as observed in the present study. Additionally, the SML study identified other factors such as the emotional burden of being diagnosed, fear and despair by survival statistics, and unavailability of effective treatment options, especially after relapse, as the main emotional impacts of lung cancer on QoL, followed by physical and financial impacts.

Treatment of patients with lung cancer during the current COVID-19 pandemic is particularly challenging. Among other discussed topics, the impact of COVID-19, specifically in the context of NSCLC treatment, emerged as one of the key concerns among patients/caregivers. Cancellations or delays in treatment appointments, infection concerns due to being vulnerable, inability to find the PPE needed for safety, failure to adhere to COVID-19 safety requirements during treatment administration, and lack of aftercare were some of the major concerns.

Other key unmet needs were identified as a feeling of abandonment by patients due to lack of empathy and communication, or lack of a comprehensive and easy-to-understand explanation of the process/treatment, lack of quality in effective treatment, patient care and services, insurance coverage, and prejudice against lung cancer. Our findings regarding unmet needs and improving healthcare experiences were broadly consistent with the previous qualitative research highlighting a similar need for improving healthcare experiences [31, 41].

As ICIs have moved to the front-line setting and are administered as monotherapy or in combination with chemotherapy to all NSCLC patients [10], cost has become increasingly important as the treatment is quite expensive. This has led some healthcare systems to impose a fixed duration of immunotherapy of 2 years; for instance, in the UK, immunotherapy is currently capped at 2 years by the National Health Services (NHS) [42]. The social media posts from the SML study showed that patients were worried about unavailability of treatment after 2 years, especially for cases responding well to the treatment. Unlike the UK, in countries such as Switzerland, the decision on treatment duration remains at the physician’s discretion [42]. In addition to the financial toxicity, chronic adverse events (immune- or non-immune related) may develop in treated patients, which can drive the decision to stop immunotherapy at 2 years or sooner [43]. Furthermore, long-term survival data from RCTs have demonstrated that most patients who completed 2 years of immunotherapy had a durable response in the follow-up period, and that stopping after 2 years does not appear to negatively impact survival [44–46].

Overall, the present SML study highlighted some key differences in treatment goals and language used by patients/caregivers and HCPs. The findings from this study provide an important insight into the need for enhancing physician-patient communication. Identifying patients’ expectations, unmet needs (e.g., awareness generation around disease, greater physician empathy), and treatment goals could create opportunities for optimizing health care, and in the long run, increased patient satisfaction.

### Limitations

This study is subject to the inherent limitations of any social media research, i.e., a general assumption that information provided by patients is authentic and was voluntarily shared with other patients publicly. Moreover, the quality of insights gathered from the analysis of digital conversations is dependent on the richness of those patient conversations, i.e., the details shared by patients about their health condition, including treatment, medication, disease management challenges, and QoL. Another limitation is that we have analyzed the overall results without splitting them into late and early stages of the disease and this may have an impact on treatment patterns, sequencing, and, potentially, QoL. Due to the unstructured nature of social data, it was not always possible to find information for every research question. An objective evaluation was carried out to determine if the available information supported a research question or not, and findings were reported accordingly. The unstructured nature of the data also leads to variation in sample size related to each key performance indicator. The nature of available digital content varied by platform, primarily due to format (e.g., Twitter versus forums), and the most appropriate platform to derive in-depth consultative insights was determined during the sampling process. The reduced sample size made interpretation of local results difficult; for instance, a small number of posts might have led to a random finding while analyzing differences between the countries.

## Conclusions

This SML approach offers a unique way of generating real-world evidence that complements the data generated through traditional non-interventional studies. Despite some limitations inherent in social listening, this study provides valuable insights into the lung cancer experience and highlights patients’ desire to increase public awareness and for greater physician empathy. To conclude, understanding patient treatment goals and adopting patient-friendly language could improve communication and collaboration between HCPs and their patients, which in turn, could inform how HCPs can provide the kind of comprehensive and holistic healthcare support patients need. More work is needed to explore stakeholder perceptions outside the pandemic situation, and with new treatment options (e.g., chemo-immunotherapy should be avoided with new combinations of immuno-immunotherapies). Access to treatment options should also be considered since this varies from country to country.

## Supplementary Information

**Additional file 1: Supplementary Table 1.** Social media search strings. **Supplementary Table 2.** Pre-defined inclusion/exclusion criteria. **Supplementary Figure 1.** Three tier process for data analysis. **Supplementary Figure 2.** Key conversation themes by country. **Supplementary Text.** Mentions of the cancer type and stage, metastatic sites, and genetic evaluation. **Supplementary Figure 3.** a) Cancer type; b) Cancer stage; c) Metastasised sites; d) Genetic evaluation. **Supplementary Figure 4.** Key treatment types by sentiment. **Supplementary Figure 5.** Treatment by genetic targets. **Supplementary Figure 6.** Treatment features. **Supplementary Figure 7.** Key unmet needs. a) Discussion around the impacts of COVID-19; b) Other key unmet needs.

## Data Availability

All data used in this study were retrieved from publicly available sources. The raw datasets generated and/or analyzed during the current study are not publicly available to avoid participant identification but are available from the corresponding author on reasonable request, from qualified external researchers. These requests are reviewed and approved by an independent review panel on the basis of scientific merit.

## Abbreviations

ALK: Anaplastic lymphoma kinase
BRAF: v-raf murine sarcoma viral oncogene homolog B1
EMA: European medicines agency
EGFR: Epidermal growth factor receptor
FDA: Food and Drug Administration
HCPs: Healthcare professionals
ICIs: Immune checkpoint inhibitors
KRAS: Kirsten rat sarcoma viral oncogene homolog
MET: N-methyl-N′-nitroso-guanidine human osteosarcoma transforming gene
NHS: National Health Service
NSCLC: Non-small cell lung cancer
NTRK: Neurotrophic tyrosine receptor kinase
PD-1: Programmed cell death-1 receptor
PPEs: Personal protective equipment
PROs: Patient-reported outcomes
QoL: Quality of life
RCTs: Randomized controlled trials
RET: Rearranged during transfection
ROS1: C-ros oncogene 1
RWD: Real-world databases
SCLC: Small cell lung cancer
SML: Social media listening
