# Supplementary material for: Understanding the lived experience of lung cancer: a European social media listening study
Source: BMC Cancer. 2022 Apr 30;22:475. doi: 10.1186/s12885-022-09505-4 (PMC9055221; doi:10.1186/s12885-022-09505-4)
Supplement: Supplementary file 1 — Additional file 1: Supplementary Table 1. Social media search strings. Supplementary Table 2. Pre-defined inclusion/exclusion criteria. Supplementary Figure 1. Three tier process for data analysis. Supplementary Figure 2. Key conversation themes by country. Supplementary Text. Mentions of the cancer type and stage, metastatic sites, and genetic evaluation. Supplementary Figure 3. a) Cancer type; b) Cancer stage; c) Metastasised sites; d) Genetic evaluation. Supplementary Figure 4. Key treatment types by sentiment. Supplementary Figure 5. Treatment by genetic targets. Supplementary Figure 6. Treatment features. Supplementary Figure 7. Key unmet needs. a) Discussion around the impacts of COVID-19; b) Other key unmet needs. [file 12885_2022_9505_MOESM1_ESM.docx]

**SUPPLEMENTARY MATERIAL**

**Understanding the Lived Experience of Lung Cancer: A European Social Media Listening Study**

**Authors:** Ana Rodrigues^1^, Jyoti Chauhan^2^, Alexandros Sagkriotis^3^, Sathyaraj Aasaithambi^2^, Michele Montrone^4^

**Affiliations:** ^1^Medical Oncology, Instituto Português de Oncologia do Porto Francisco Gentil, EPE, Porto, Portugal; ^2^Novartis Healthcare Pvt Ltd (H.A.), Hyderabad, India; ^3^Novartis Pharma AG, Basel, Switzerland; ^4^Medical Thoracic Oncology Unit, IRCCS Istituto Tumori “Giovanni Paolo II”, Bari, Italy

**CONTENTS**

**Supplementary Table 1** Social media search strings[.................................................................2](#_Toc65855676)

**Supplementary Table 2** Pre-defined inclusion/exclusion criteria...............................................8

**Supplementary Figure 1** Three tier process for data analysis....................................................8

**Supplementary Figure 2** Key conversation themes by country..................................................9

**Supplementary Text** Mentions of the cancer type and stage, metastatic sites, and genetic evaluation ....................................................................................................................................9

**Supplementary Figure 3 a)** Cancer type; **b)** Cancer stage; **c)** Metastasised sites; **d)** Genetic evaluation..................................................................................................................10

**Supplementary Figure 4** Key treatment types by sentiment.....................................................11

**Supplementary Figure 5** Treatment by genetic targets.............................................................12

**Supplementary Figure 6** Treatment features............................................................................12

**Supplementary Figure 7** Key unmet needs. **a)** Discussion around the impacts of COVID-19; **b)** Other key unmet needs...........................................................................................................13

**Supplementary Table 1. Social media search strings**

| \| **Language** \| **Search terms used** \| \| --- \| --- \| \| **Danish** \| ((luftrør* NEAR/3 kræft*) OR ((bronch* OR luftvej*) NEAR/3 kræft*) OR ((lungesæk* OR luftsæk* OR alveol*) NEAR/3 kræft*) OR "Lung cancer*" OR Lungcancer* OR #lungcancer OR Lungekræft* OR #Lungekræft OR lungecarcinom* OR lungekarcinom* OR ((Lung* OR LCC OR GCCL) NEAR/4 (Kræft* OR cancer* OR carcinom* OR karcinom*)) OR NSCLC OR #NSCLC OR "#MetastaticNSCLC" OR "MetastatiskNSCLC" OR "mNSCLC" OR "#mNSCLC" OR "#mnsclc" OR "#MetastaticLungCancer" OR "Storcellet lungekarsinom" OR ((Lung* OR NSCLC OR "#NSCLC" OR "#nsclc" OR LCC OR GCCL) NEAR/4 (adenocarcinom* OR pladecellecarcinom* OR storcellekarcinom* OR adenokarcinom* OR pladecellekarcinom* OR storcellecarcinom*)) OR ((Lung* OR NSCLC OR "#NSCLC" OR "#nsclc" OR LCC OR GCCL OR neuroend*) NEAR/4 (karcinom* OR carcinom*) NEAR/4 ("squamous cell" OR plateepitel* OR pladecelle* OR storcelle* OR "stor celle*" OR "large cell")) OR ((adenosquamous OR adenosquamøs* OR sarcomatoid* OR sarkomoid*) NEAR/4 (carcinom* OR karsinom* OR adenokarcinom* OR adenocarcinom*))) NOT (SCLC OR "#sclc" OR "oat cell cancer" OR "havre celle kræft" OR havrecelle*) \| \| **Dutch** \| (("kanker aan de luchtpijp" OR "kanker van de luchtpijp" OR "luchtpijpkanker" OR "luchtpijp kanker" OR "kanker van de bronchiën" OR "kanker aan de bronchiën" OR "kanker van de luchtpijpvertakkingen" OR "bronchiale kanker" OR "kanker van de longblaasjes" OR "kanker van longblaasjes" OR "kanker aan de longblaasjes" OR "kanker van de pulmonaire alveolen" OR "Long kanker" OR longkanker OR #longkanker OR #LongKanker OR #Longkanker OR (Long* NEAR/4 kanker) OR "niet-kleincellige longkanker" OR "niet kleincellige long kanker" OR "niet-kleine-cellen longkanker" OR "Niet-kleincellig longkanker" OR "Niet-kleincellige long carcinoom" OR "Niet-kleincellige longcarcinoom" OR "Niet kleincellige long carcinoom" OR NSCLC OR #NSCLC OR #nsclc OR "#MetastatischeNSCLC" OR "#Metastatischensclc" OR Metastatischnsclc OR "MetastatischNSCLC" OR "mNSCLC" OR "#mNSCLC" OR "#mnsclc" OR mNSCLC OR "#MetastatischeLongKanker" OR "#metastatischelongkanker" OR "#Metastasenlongkanker" OR "longkanker met metastasen" OR "#UitgezaaideLongkanker" OR ((Long* OR NSCLC OR "#NSCLC" OR "#nsclc") NEAR/4 (adenocarcinoom OR plaveiselcelcarcinoom OR "grootcellig carcinoom" OR "grootcellig ongedifferentieerd carcinoom" OR "adeno carcinoom" OR "sarcomatoïde carcinoom"))) NOT (SCLC OR "#SCLC" OR "#sclc" OR "havercel kanker" OR "haver cel kanker" OR "havercel carcinoom" OR "haver cel carcinoom")) \| \| **English** \| (("Cancer of lung air sacs" OR "Cancer of bronchi" OR "Cancer of trachea"OR "Lung cancer" OR Lungcancer OR #Lungcancer OR #lungcancer OR #LungCancer OR (Lung* NEAR/4 cancer) OR "non-small cell lung cancer" OR "non small cell lung cancer" OR "non-small-cell lung cancer" OR "Non-Small-Cell Lung Cancer" OR "Non-small-cell lung carcinoma" OR "Non-small cell lung carcinoma" OR "Non small cell lung carcinoma" OR NSCLC OR #NSCLC OR #nsclc OR "#MetastaticNSCLC" OR "#Metastaticnsclc" OR Metastaticnsclc OR "MetastaticNSCLC" OR "mNSCLC" OR "#mNSCLC" OR "#mnsclc" OR mNSCLC OR "#MetastaticLungCancer" OR "#metastaticlungcancer" OR "#Metastaticlungcancer" OR "#MetastaticLungcancer" OR ((Lung* OR NSCLC OR "#NSCLC" OR "#nsclc") NEAR/4 (adenocarcinoma* OR "squamous cell carcinoma*" OR "large cell carcinoma*" OR "large cell neuroendocrine carcinoma" OR "adenosquamous carcinoma" OR "sarcomatoid carcinoma"))) NOT (SCLC OR "#SCLC" OR "#sclc" OR "oat cell cancer" OR "oat-cell cancer" OR "oat-cell carcinoma" OR "oat cell carcinoma")) \| \| **Finnish** \| (("henkitorven syöpä" OR ((keuhkoputki* OR keuhkorakkul*) NEAR/3 (syöpä* OR syövä* OR syöpi*)) OR keuhkosyöpä OR keuhkosyöpä* OR keuhkosyövän OR keuhkosyövät OR keuhkosyöpi* OR keuhkosyövä* OR keuhkosyövi* OR #keuhkosyöpä OR (keuhko* NEAR/4 (syöpä OR syöpä* OR syövä* OR syöpi*)) OR “ei-pienisoluinen keuhkosyöpä” OR “ei-pienisoluisen keuhkosyövän” OR “ei-pienisoluisia keuhkosyövän” OR “ei-pienisoluisen keuhkosyövässä” OR “ei-pienisoluisiin keuhkosyöpiin” OR “ei-pienisoluista keuhkosyöpää” OR “ei-pienisoluiseen keuhkosyöpään” OR “ei-pienisoluisessa keuhkosyövässä” OR NSCLC OR #NSCLC OR #nsclc OR “#MetastaattinenNSCLC” OR “etäpesäkkeinenNSCLC” OR “mNSCLC” OR “mnsclc” OR mNSCLC OR “etäpesäkkeinen keuhkosyöpä” OR “metastaattinen keuhkosyöpä” OR “metastaatti* keuhkosyöpä*” OR “metastaatti* keuhkosyövä* ” OR “metastas* keuhkosyöpä*” OR “metastas* keuhkosyövä*” OR ((keuhko* OR NSCLC OR "#NSCLC" OR "#nsclc") NEAR/4 (adenocarcinom* OR levyepiteelisyöpä* OR okasolusyöpä* OR levyepiteelisyövä* OR levyepiteelisyövi* OR levyepiteelikarsinooma OR levyepiteelikarsinooma* OR okasolusyövä* OR okasolusyövi* OR “suurisoluinen keuhkosyöpä” OR “suurisoluinen syöpä” OR “suurisoluinen karsinooma” OR “suurisoluinen neuroendokriininen karsinooma” OR adenoskvamoosikarsinooma OR adenoskvamoosikarsinoom* OR “sarkomatoidi karsinooma”))) NOT (“pienisoluinen keuhkosyöpä” OR “pienisoluiset keuhkosyövät” OR “pienisoluis* keuhkosyövä*” OR “pienisoluis* keuhkosyöpä*” OR SCLC OR "#SCLC" OR "#sclc" OR “kaura-solusyöpä” OR “kaura-solukarsinooma*” OR kaurasolukarsinooma*)) \| \| **French** \| (("cancer des sacs aériens pulmonaires" OR "cancer des sacs d'air pulmonaires" OR "cancer de sacs d'air des poumons" OR "Cancer de sacs aériens des poumons" OR "Cancer des alvéoles pulmonaires" OR "cancer des bronches" OR "cancer bronchique" OR "cancer du poumon" OR "cancer des poumons" OR #cancerpoumon OR #cancerdupoumon OR (poumon* NEAR/4 cancer) OR "cancer du poumon non à petites cellules" OR "cancer du poumon non-à-petites cellules" OR "cellules non-petites de carcinomes pulmonaires" OR "carcinome pulmonaire non à petites cellules" OR "carcinome pulmonaire humain à cellules non petites" OR NSCLC OR #NSCLC OR #MetastaticNSCLC OR métastatique OR "Cancer du poumon métastatique" OR #metastatique OR #MetastaticLungCancer OR ((poumon* OR NSCLC OR #NSCLC) NEAR/4 (adénocarcinome* OR "carcinome squameux*" OR "carcinome à grandes cellules*" OR "carcinome neuroendocrinien à grandes cellules" OR "carcinome adénosquameux" OR "carcinome sarcomatoïde"))) NOT (#SCLC OR SCLC OR "carcinone à cellules en grains d'avoine")) \| \| **German** \| (("Krebs des Lungensacks" OR Bronchialkrebs OR Bronchialkarzinom OR Luftröhrenkrebs OR Tracheakarzinom OR Trachealkarzinom OR "Krebserkrankung der Luftröhre" OR Luftröhrenkrebs OR Tracheakarzinom OR Trachealkarzinom OR "Krebserkrankung der Luftröhre" OR Bronchialkrebs OR Bronchialkarzinom OR "Krebs des Lungensacks" OR Lungenkrebs OR #Lungenkrebs OR (Lunge* NEAR/4 Krebs) OR "nicht kleinzelliger Lungenkrebs" OR "nicht-kleinzelliger Lungenkrebs" OR "nicht kleinzelliger Krebs" OR "Nicht-kleinzelliges Lungenkarzinom" OR "Nicht kleinzelliges Lungenkarzinom" OR "Nicht-kleinzelliges Bronchialkarzinom" OR "Nicht kleinzelliges Bronchialkarzinom" OR NSCLC OR #NSCLC OR #nsclc OR "#MetastaticNSCLC" OR "metastasierender NSCLC" OR mNSCLC OR "#mNSCLC" OR "metastatischer Lungenkrebs" OR "gestreuter Lungenkrebs" OR "#metastatischerlungenkrebs" OR "#Metastat" OR ((Lunge* OR NSCLC OR "#NSCLC" OR "#nsclc") NEAR/4 (Adenokarzinom* OR Drüsenkrebs OR Plattenepithelkarzinom* OR Großzellkarzinom* OR "Großzelliges neuroendokrines Karzinom" OR Adenosquamkarzinom* OR "sarkomatoides Karzinom"))) NOT (SCLC OR "#SCLC" OR Haferzellkrebs OR Haferzellkarzinom)) \| \| **Italian** \| (("Tumore agli alveoli" OR "tumore degli alveoli" OR "cancro degli alveoli" OR "Tumore ai bronchi" OR "carcinoide bronchiale" OR "cancro ai bronchi" OR "Cancro alla trachea" OR "tumore della trachea" OR "cancro a* polmon*" OR "cancro de* polmon*" OR "carcinom* polmonar*" OR "tumor* polmonar*" OR "tumor* a* polmon*" OR "tumor* de* polmon*" OR #cancropolmone OR #cancropolmoni OR #cancrodelpomone OR #cancrodeipolmoni OR #cancroalpolmone OR #cancroaipolmoni OR #carcinomapolmonare OR #tumorepolmonare OR #tumorepolmoni OR #tumorepolmone OR #tumoredelpolmone OR (polmon* NEAR/4 cancro) OR "cancro de* polmon* non a piccole cellule" OR "cancro a* polmon* non a piccole cellule" OR "tumore polmonare non a piccole cellule" OR "tumore de* polmon* non a piccole cellule" OR "tumore a* polmon* non a piccole cellule" OR "carcinoma polmonare non a piccole cellule" OR "carcinoma non-microcitoma" OR "carcinoma polmonare non microcitoma" OR "carcinoma polmonare non microcitico" OR NSCLC OR #NSCLC OR #nsclc OR "#NSCLCmetastatico" OR "#nsclcmetastatico" OR "NSCLC metastatico" OR "#mNSCLC" OR "#mnsclc" OR "#CancroPolmoneMetastatico" OR "#TumorePolmonareMetastatico" OR "#CarcinomaPolmonareMetastatico" OR ((polmon* OR NSCLC OR "#NSCLC" OR "#nsclc") NEAR/4 (adenocarcinom* OR "carcinom* a cellule squamose" OR "carcinom* squamocellular*" OR "tumor* a cellule squamose" OR "carcinom* squamos*" OR "carcinom* a grandi cellule" OR "carcinom* neuroendocrin* a grandi cellule" OR "carcinom* adenosquamos*" OR "carcinom* sarcomatoid*"))) NOT (SCLC OR "#SCLC" OR "#sclc" OR "tumore a chicco d'avena" OR "cancro a chicco d'avena" OR "carcinoma a chicco d'avena")) \| \| **Norwegian** \| ((luftrør* NEAR/3 kreft*) OR ((bronk* OR bronch*) NEAR/3 kreft*) OR ((lungesug* OR alveol*) NEAR/3 kreft*) OR "Lung cancer*" OR Lungcancer* OR #lungcancer OR Lungekreft* OR #lungekreft OR lungekarsinom* OR ((Lung* OR LCC OR GCCL) NEAR/4 (kreft* OR cancer* OR karsinom* OR carcinom*)) OR NSCLC OR #NSCLC OR #nsclc OR "#MetastaticNSCLC" OR "#Metastaticnsclc" OR Metastaticnsclc OR "MetastatiskNSCLC" OR "mNSCLC" OR "#mNSCLC" OR "#mnsclc" OR "#MetastaticLungCancer" OR "#metastaticlungcancer" OR "Storcellet lungekarsinom" OR ((Lung* OR NSCLC OR "#NSCLC" OR "#nsclc" OR LCC OR GCCL) NEAR/4 (adenokarsinom* OR plateepitelkarsinom* OR Storcellekarsinom* OR Kjempecellekarsinom)) OR ((Lung* OR NSCLC OR "#NSCLC" OR "#nsclc" OR LCC OR GCCL OR nevroendokrin* OR neuroendocrine) NEAR/4 (karsinom* OR carcinom*) NEAR/4 ("squamous cell" OR plateepitel* OR storcelle* OR "large cell")) OR ((adenosquamous OR adenosquamøs* OR sarcomatoid* OR sarkomoid*) NEAR/4 (carcinom* OR karsinom* OR adenokarsinom*))) NOT (SCLC OR "#sclc" OR "oat cell cancer" OR "havre celle" OR havrecelle*) \| \| **Portuguese** \| (("Cancro do Pulmão" OR "Cancro nos Pulmões" OR "Cancro do Pulmao" OR "Cancro nos Pulmoes" OR "Cancro da Traqueia" OR "Cancro dos Brônquios" OR "Cancro Pulmonar" OR #Cancropulmão OR #cancropulmao OR (Pulmão* NEAR/4 cancro) OR "cancro do pulmão de não-pequenas células" OR "carcinomas pulmonares de não pequenas células" OR CPNPC OR #CPNPC OR #cpnpc OR "CPNPC metastático" OR "cpnpc metastático" OR "#CPNPCmetastatico" OR "#cpnpcmetastático" OR "CPNPCm" OR "cpnpcm" OR "#CancroMetastaticodoPulmao" OR "#cancrometastaticodopulmao" OR "#Cancrometastaticodopulmao" OR "#cancrometastaticodoPulmao" OR ((Pulmão* OR CPNPC OR #CPNPC OR "#cpnpc") NEAR/4 (adenocarcinoma* OR "carcinoma escamoso*" OR "carcinoma adenoscamoso*" OR "carcinoma grandes celulas*" OR "carcinomas de células grandes" OR "cancro pulmonar de grandes células" OR "carcinomas pulmonares de grandes células" OR "carcinoma pulmonar de não pequenas celulas" OR "carcinoma neuroendócrino" OR "carcinoma neuroendrócrino grandes celulas" OR "carcinoma adeno escamoso" OR "carcinoma sarcomatóide"))) NOT ("carcinoma pulmonar de pequenas celulas" OR CPPC OR #CPPC OR #cppc OR "CPPC metastático" OR "cppc metastático" OR "CPPC Metastático")) \| \| **Spanish** \| (("cancer de alveolos pulmonares" OR "cancer en los alveolos pulmonares" OR "cancer de bronquios" OR "cancer bronquial" OR "cancer de traquea" OR "neoplasia traqueal primaria" OR "cancer de pulmon" OR cancerdepulmon OR #cancerdepulmon OR (pulmon NEAR/4 cancer) OR "carcinoma pulmonar no microcitico" OR “cancer pulmonar de celulas no pequeñas” OR CPNM OR #CPNM OR #cpnm OR (CPNM NEAR/6 Metastasi) OR #ConvivirConCancerDepulmon OR “#Cancerdepulmonmetastasico" OR ((pulmon OR CPNM OR "#CPNM" OR "#cpnm") NEAR/4 (adenocarcinoma OR "Carcinoma espinocelular*" OR “epitelioma espinocelular” OR espinalioma OR "carcinoma* pulmonar de celulas grandes" OR “celulas grandes” OR "carcinoma sarcomatoide"))) NOT (SCLC OR "#SCLC" OR "#sclc" OR #Elbloqueoesreal OR #tueresigc OR #pezfueradelagua OR #epoc OR "amancio ortega" OR #amancioortega)) AND sourcegeo_accuracy:high \| \| **Swedish** \| ((((((lungluftsäck* OR alveol*) NEAR/3 cancer*) OR bronk* OR bronch*) NEAR/3 cancer*) OR luftstrupencancer OR (luftstrup* NEAR/3 cancer*) OR "Lung cancer" OR Lungcancer OR #Lungcancer OR #lungcancer OR #LungCancer OR (Lung* NEAR/4 cancer) OR "icke-småcellig lungcancer" OR " ickesmåcellig lungcancer" OR "icke-småcellig-lungcancer" OR "icke-små-cellig lungcancer" OR "Icke-småcellig lungkarcinom" OR "Icke-små-cellig lungkarcinom" OR "Icke-småcellig-lungkarcinom" OR NSCLC OR #NSCLC OR #nsclc OR “#metastiskNSCLC” OR “#metastisknsclc” OR metastisknsclc OR metastiskNSCLC OR “#MetastatiskLungCancer” OR "#metastatisklungcancer” OR "#Metastatisklungcancer” OR ((Lung* OR NSCLC OR "#NSCLC" OR "#nsclc") NEAR/4 (adenokarcinom* OR "skivepitelcancer*" OR "storcellscancer*" OR "neuroendokrin karcinom i stor cell" OR "neuroendokrin karcinom i stor cell" OR “neuroendokrin karcinom i storcell” OR "sarcomatoid karcinom"))) NOT (SCLC OR "#SCLC" OR "#sclc" OR "havrecellcancer" OR “havre cell cancer” OR “havre cellcancer” OR “havre cellcancer” OR "havre-cellkarcinom" OR “havrecell karcinom” OR havrecellkarcinom)) \| |
| --- | --- | --- | --- | --- | --- | --- | --- | --- | --- | --- | --- | --- | --- | --- | --- | --- | --- | --- | --- | --- | --- | --- | --- | --- |

**Supplementary Table 2.** Pre-defined inclusion/exclusion criteria

| \| **Inclusion criteria** \| Posts INCLUDED in the final analysis:   - Contained information about lung cancer and NSCLC; specifically causes, symptoms experienced, diagnosis, treatment, management, disease endpoint (remission, recurrence, death), QoL impact, and unmet needs \| \| --- \| --- \| \| **Exclusion criteria** \| Posts EXCLUDED in the final analysis:   - Contained buy/sell content or market reports - Contained animal content - Were job postings - Were link duplicates - Only contained non-insightful content; simple mentions of lung cancer or NSCLC without meaningful insight \| |
| --- | --- | --- | --- | --- |
| NSCLC, non-small cell lung cancer; QoL, quality of life |

**Supplementary Figure 1.** Three tier process for data analysis

| *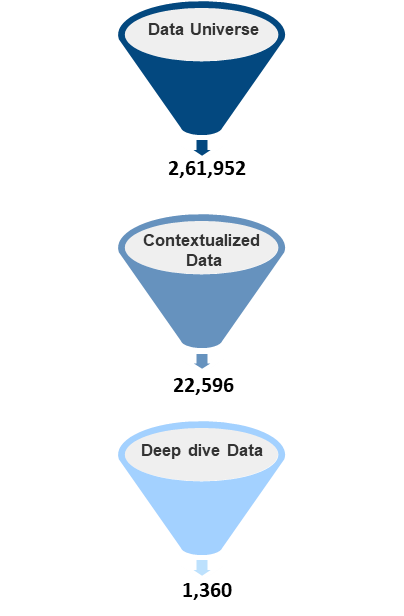*  **~1360** filtered data points from key stakeholders including patients, caregivers, HCPs, PSGs, and others. These conversations specifically help us in deep dive analysis on in scope KBQs  **~22,600** conversations providing insights around patient journey stages and other patient-centric topics. Deep dive on treatment-related discussions  **~262,000** overall mentions of lung cancer and NSCLC |
| --- |
| HCPs, healthcare professionals; KBQs, knowledge-based queries; NSCLC, non-small cell lung cancer; PSGs, patient support groups. |

**Supplementary Figure 2.** Key conversation themes by country

| 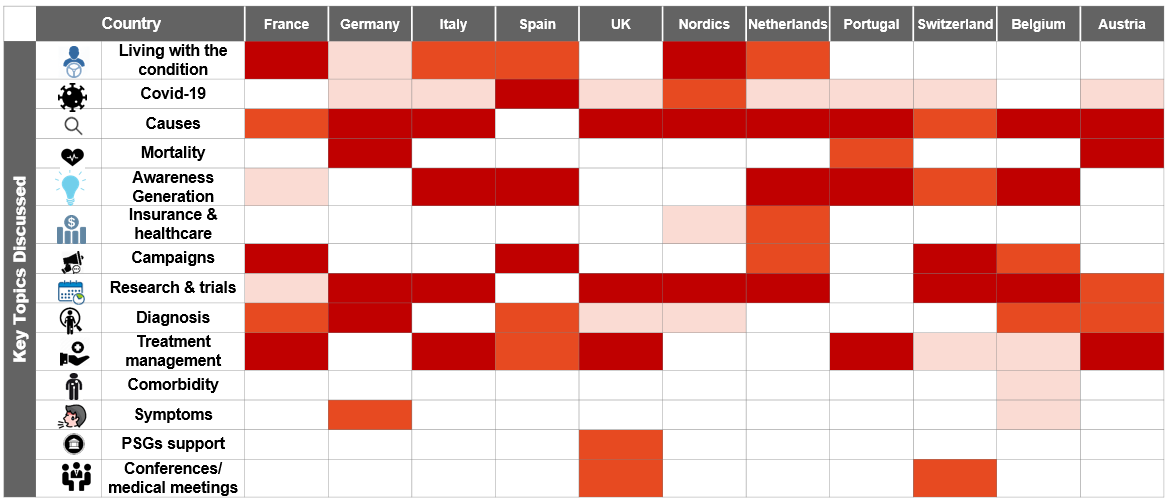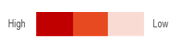 |
| --- |
| A non-shaded box indicates that no conversations were identified for that particular topic in that country |

**Supplementary section.** *Mentions of the cancer type and stage, metastatic sites, and genetic evaluation*

A number of posts discussed cancer type (n = 1360); however, general mentions of lung cancer were the most common (75%), followed by NSCLC (23%). Patients/caregivers mostly discussed the condition as “lung cancer”, whereas the term “NSCLC” was mostly used by specialist news feeds or HCPs. Out of 550 conversations on cancer stages, almost 91% had late-stage cancer, 11% had early-stage cancer, and 8% had stage III cancer. However, occasionally the terminology varied among the posts, resulting in an overlap of figures (**Supplementary Figure 3).**

Many stakeholders did not specify metastatic sites, they just mentioned that their cancer had metastasized. When discussed (n = 188), brain (47%), bone (20%), and lymph nodes (16%) were the most frequently mentioned metastatic sites (**Supplementary Figure 3).** Owing to a lack of awareness around the topic, mentions of genetic evaluation were very low across all countries (n = 13).

**Supplementary Figure 3. a)** Cancer type **b)** Cancer stage; **c)** Metastasised sites; **d)** Genetic evaluation

| 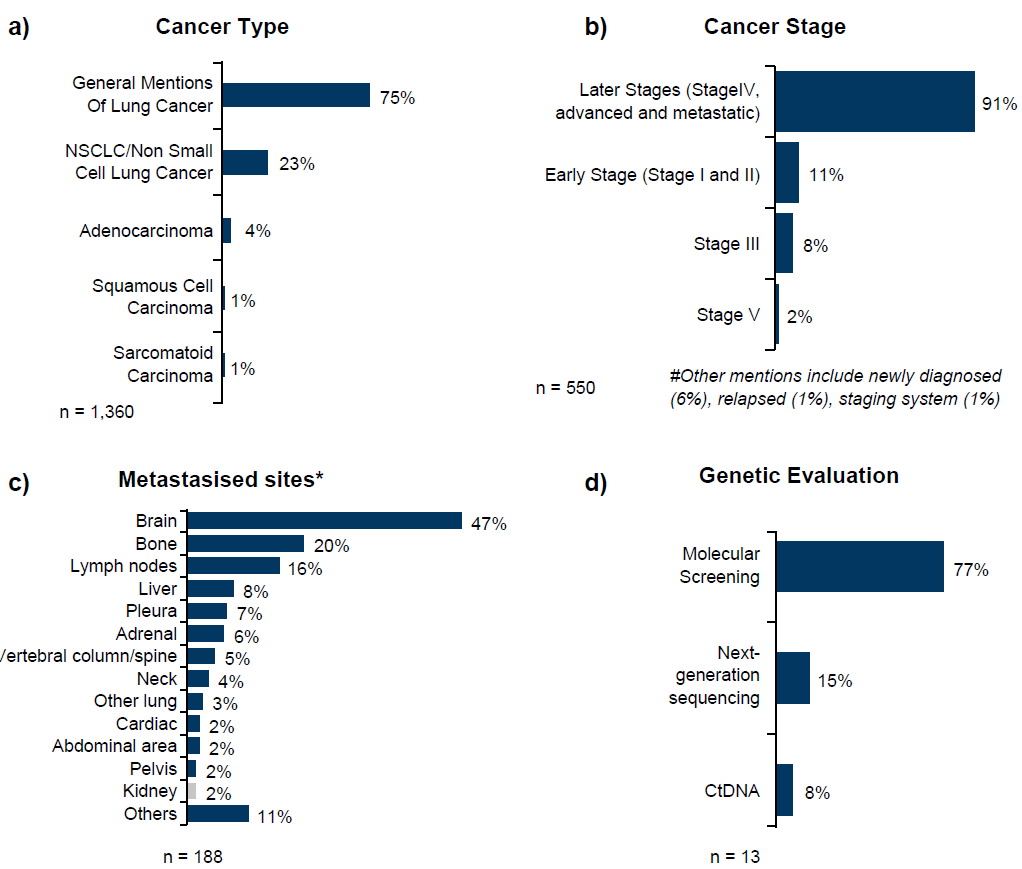 |
| --- |
| * Total % may be >100% due to mention of multiple themes in posts.  CtDNA, circulating tumor DNA |

**Supplementary Figure 4.** Key treatment types by sentiment

| 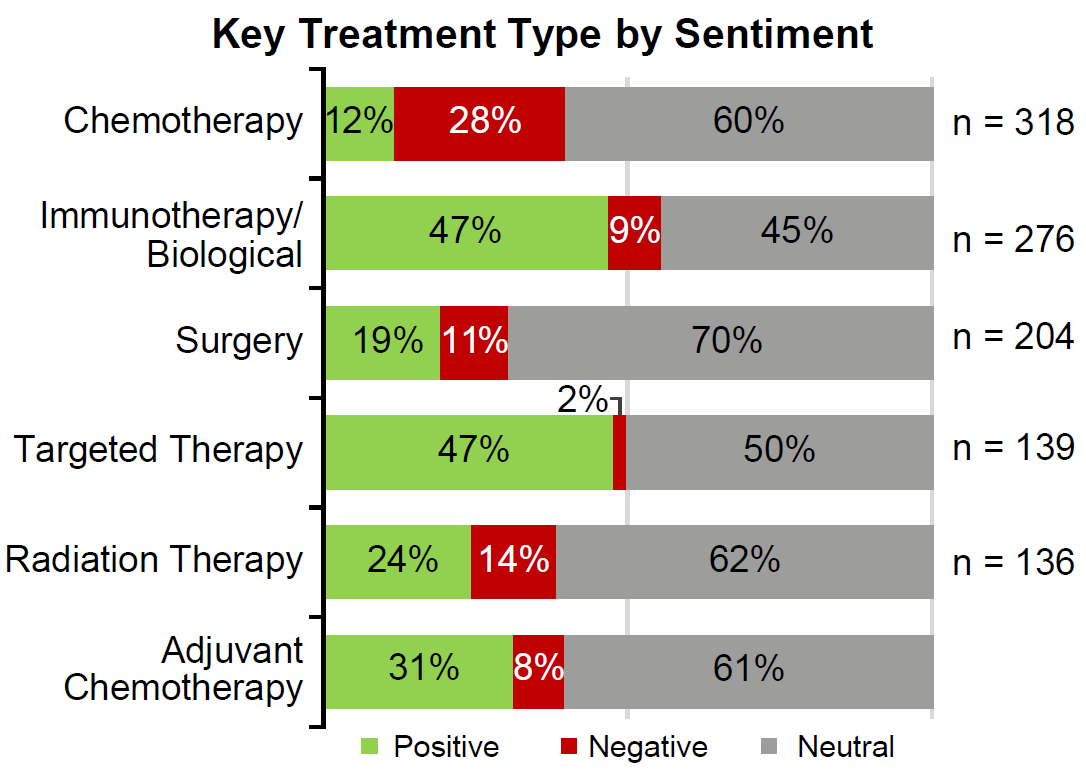 |
| --- |

**Supplementary Figure 5.** Treatment by genetic targets

| 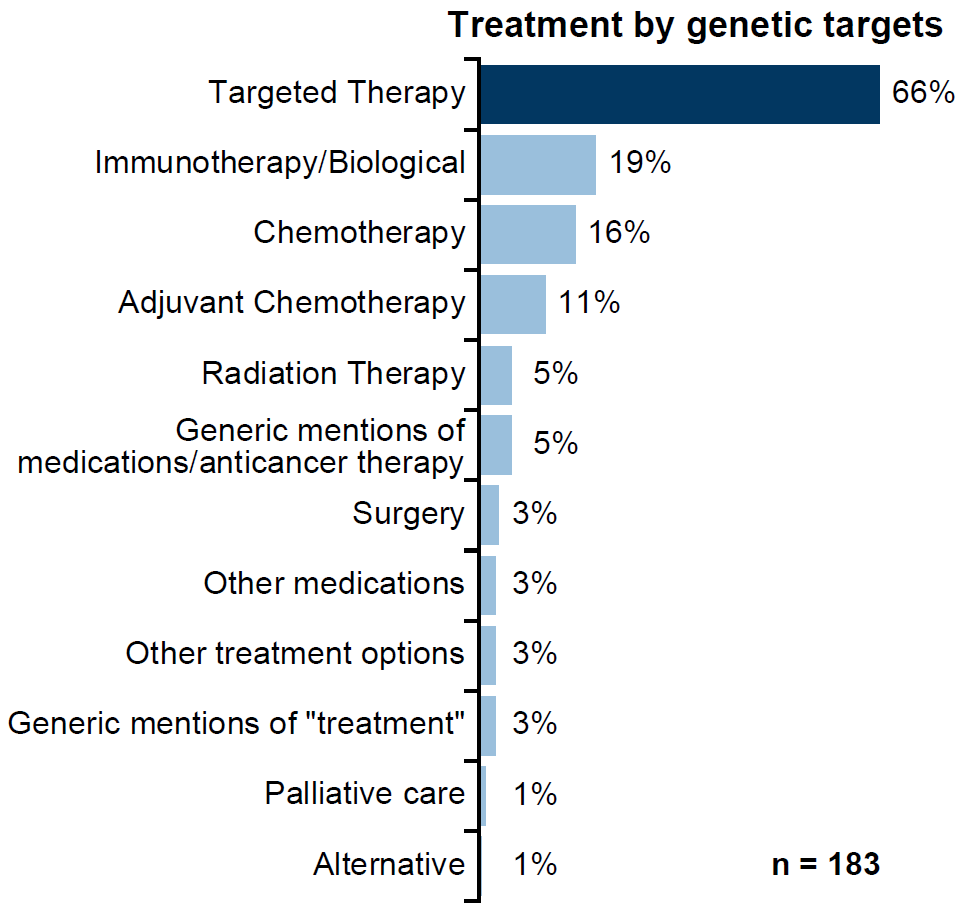 |
| --- |

**Supplementary Figure 6.** Treatment features

| 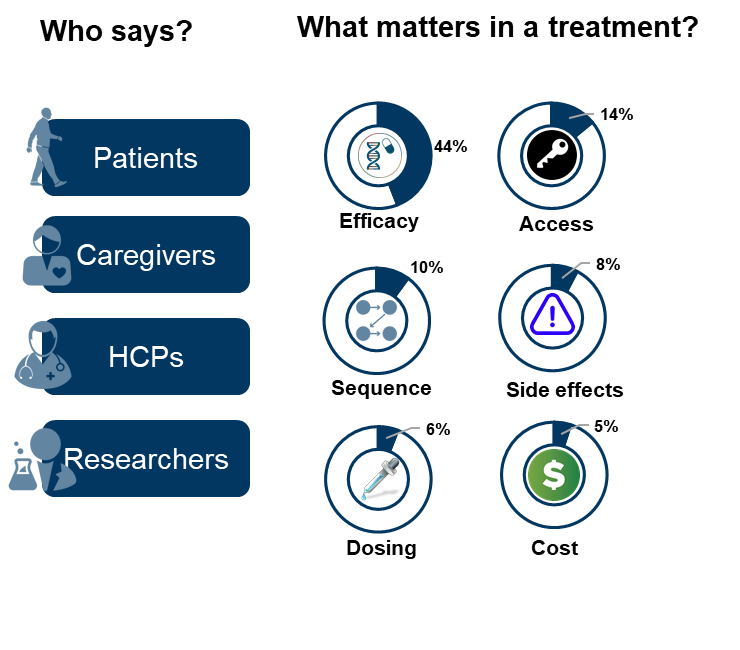 |
| --- |
| HCPs, healthcare professionals. |

**Supplementary Figure 7.** Key unmet needs. **a)** Discussion around the impacts of COVID-19; **b)** Other key unmet needs

| 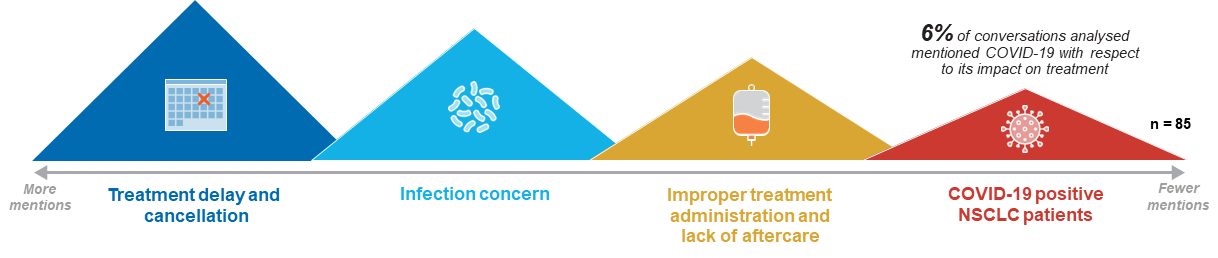  **b)**  **a)**  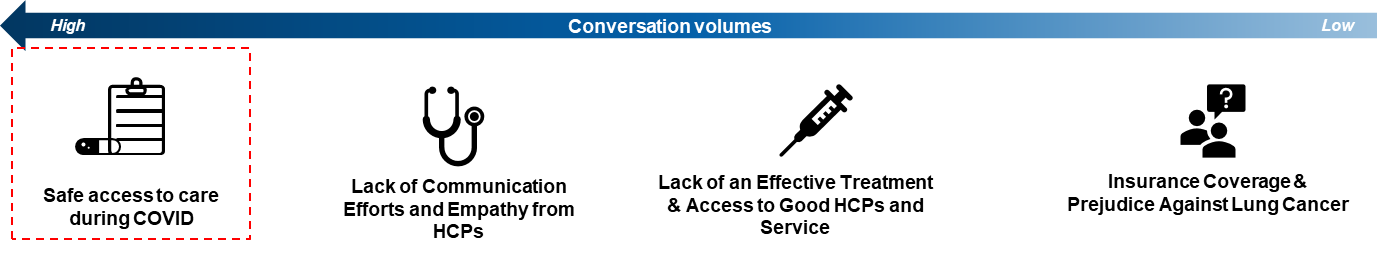 |
| --- |
| COVID-19, coronavirus disease 2019; HCPs, healthcare professionals; NSCLC, non-small cell lung cancer. |
